# An Evidence-Based Update on Fixation Procedures for Acute and Chronic Osteochondral Lesions of the Talus

**DOI:** 10.1177/19476035241280072

**Published:** 2024-09-23

**Authors:** Tomoyuki Nakasa, Yasunari Ikuta, Naoki Haraguchi, Chul Hyun Park, Christian David Weber, Quinten G.H. Rikken, Jari Dahmen, Sjoerd A.S. Stufkens, Gino M.M.J. Kerkhoffs, Masato Takao

**Affiliations:** 1 Department of Orthopaedic Surgery, Graduate School of Biomedical and Health Sciences, Hiroshima University, Hiroshima, Japan; 2 Department of Orthopaedic Surgery, St. Marianna University Yokohama Seibu Hospital, Yokohama, Japan; 3 Department of Orthopaedic Surgery, College of Medicine, Yeungnam University, Gyeongsan, Republic of Korea; 4 Department of Orthopaedic, Trauma, and Reconstructive Surgery, RWTH University Hospital, Aachen, Germany; 5 Department of Orthopaedic Surgery and Sports Medicine, Amsterdam Movement Sciences, Amsterdam UMC, University of Amsterdam, Amsterdam, The Netherlands; 6 Clinical and Research Institute for Foot and Ankle Surgery, Jujo Hospital, Kisarazu, Japan

**Keywords:** osteochondral lesion of the talus, subchondral bone, articular cartilage, bone marrow stimulation, osteochondral fragment, fixation

## Abstract

Osteochondral lesions of the talus (OLT) involve the subchondral bone and the overlying articular cartilage. Various surgical treatments for these lesions are available, such as bone marrow stimulation (BMS), autologous osteochondral grafting, and fixation of an osteochondral fragment. Treatment choice depends on the condition of the lesion, which includes lesion size, morphology, location, and the presence of cysts. Among the surgical procedures available to date, *in situ* fixation of the osteochondral fragment has the advantage of restoring the articular surface while preserving the native hyaline cartilage and its subchondral bone. Fixation for OLT has been shown to be clinically successful for the treatment of both acute and chronic lesions. Moreover, the indication for osteochondral fragment fixation is expanding as recent studies have found good clinical outcomes in relatively small-sized lesions. The present article describes the current evidence on fixation for acute and chronic OLT.

## Introduction

Osteochondral lesions of the talus (OLT) involve the subchondral bone and the overlying articular cartilage. OLT often interfere with the functional abilities of patients due to complaints, such as pain during or after (weightbearing) activities, swelling, and/or locking.^1^ It is thought that OLT are caused by several etiological factors, but they are mainly associated with trauma.^2^ Unstable symptomatic osteochondral fragments caused by acute trauma or repetitive (micro) trauma require surgical treatment. Surgical treatment for an OLT can be a regenerative procedure, such as bone marrow stimulation (BMS), a repair procedure such as fixation of the osteochondral fragment, or a replacement procedure such as autograft or allograft osteochondral transplantation and autologous chondrocyte implantation. The choice of treatment depends on the lesion size, location, and extent of cartilage and subchondral bone damage. Among these surgical procedures, fixation of the osteochondral fragment has the advantage of restoring the articular cartilage surface, while preserving the native hyaline cartilage and subchondral bone, and has been shown to be a successful technique in the treatment of both acute and chronic lesions.^3^ This review describes an evidence-based update of the current literature and international perspective on fixation for OLT.

## Indication

### Lesion Size

The indication for fixation is established when one can obtain rigid fixation of a symptomatic osteochondral fragment with an intact cartilage surface, regardless of lesion chronicity. In an International Consensus Meeting on Cartilage Repair of the Ankle, 91% of participants agreed that an osteochondral fragment size of at least 10 mm is required to facilitate fixation, which is supported by several previous reports of chronic cases.^3-5^ In order to obtain a stable fixation, the osteochondral fragment should ideally be fixed at 2 separate points, to obtain both axial compression and rotational stability.^3^ Historically, the osteochondral fragment was required to be sufficiently sized in order to accommodate 2 fixation devices. In contrast, fixation in previous reports was performed on lesions less than 10 mm in diameter. Stone^6^ reported a lesion larger than 7.5 mm in his report in 1996. Kumai *et al.*^7^ performed fixation for lesions larger than 8 × 8 mm (area = 50.6 mm^2^). In the report of fixation by Haraguchi *et al.*, it is stated that the average coronal fragment size was 7.0 ± 2.6 mm (range 2-15 mm), the sagittal size 9.0 ± 3.4 mm (range 2-17 mm), and the lesion area 51.2 ± 39.9 mm^2^ (range 5-147 mm^2^). The aforementioned authors found that the correlation between the lesion area and clinical outcomes was weak (r = −0.133).^8^ When further discussing the indication for fixation of smaller (<100-150 mm^2^) lesions, one can identify overlap with the indication for BMS.^9-11^ In such cases, the theoretical advantages of fixation would make it the preferred treatment of choice. Clinical outcomes of BMS have been reported as good to excellent in the short- and mid-term follow-up, but there is the possibility that they may worsen over time due to the subchondral bone deterioration. This deterioration may be caused by the biomechanical properties of the fibrocartilage-covered defect following BMS, which is inferior to the properties of the native hyaline cartilage.^12-15^ When considering the literature on fixation, Reilingh *et al.*^5^ demonstrated that the clinical outcomes and subchondral bone healing after fixation were superior compared to BMS. A recent report by Nakasa *et al.*^16^ demonstrated that the clinical outcomes of fixation were superior to those of BMS for lesions <100 mm^2^. It is, therefore, recommended to first try fixation for osteochondral fragments, even in lesions smaller than 100 mm^2^. In cases of treatment failure or intra-operative failure of the fixation BMS can still be used as a salvage procedure.

### Lesion Chronicity

To achieve good clinical outcomes after a fixation procedure and preservation of the native hyaline cartilage union between the osteochondral fragment and its bed should be achieved. The chronicity of the lesion is an important issue because it could decrease the bone healing potential. It is believed that the fixation of an osteochondral fragment could fail in cases of chronic lesions with sclerotic borders or that additional biological healing should be induced, such as an autologous bone graft.^4,17,18^ However, good clinical outcomes have been reported even in non-acute OLT. Haraguchi *et al.*^8^ showed that good outcomes could be achieved in their series with a mean time of 33 months between the initial trauma and operation. In acute cases, symptomatic displaced fragments or non-displaced fragments in the skeletally mature patient can be fixed as soon as possible to maximize the fragment healing potential.

Since the advantage of fixation is the restoration of the articular surface with a native contour and hyaline cartilage, the articular cartilage of the osteochondral fragment should be almost intact. Fixation should, therefore, be performed before the articular cartilage degeneration progresses. Computed tomography (CT) scans can provide information about the bone condition of an osteochondral lesion. The subchondral bone plate plays an important role in cartilage metabolism, indicating that damage to the subchondral bone plate leads to the loss of proteoglycans and glycoproteins of the cartilage because it no longer supports the overlying cartilage.^19,20^ Therefore, the evaluation of the subchondral bone plate in the osteochondral fragment using CT enables the prediction of the cartilage conditions. On CT images, a normal subchondral bone plate has a high chance of having a histologically normal articular surface. OLT in which bone absorption of the bed progresses but the subchondral bone plate in the osteochondral fragment is maintained may show low cartilage degeneration. Subchondral bone sclerosis in the bed of the lesion and bone absorption in the osteochondral fragment progress remarkably, and the articular cartilage of the osteochondral fragment may point to severe degeneration of articular cartilage.^21,22^ Disruption of the subchondral bone plate in the osteochondral fragment on CT imaging is helpful in predicting cartilage degeneration. It is reported that the articular surface of the osteochondral fragment within the International Cartilage Repair Society (ICRS) grade 2 could achieve good clinical results following fixation.^22,23^ Therefore, osteochondral fragments up to ICRS grade 2 can be considered for fixation. Single-Photon Emission Computed tomography/Computed Tomography (SPECT/CT), which shows the scintigraphic osteoblastic activity, has been recognized as a promising imaging modality for OLT.^24,25^ SPECT/CT provides both anatomical information and metabolic information which reflects the viability of the lesion, including pain and bone formation.^11,13,18^

## Fixation Materials

Various fixation materials have been used for the fixation of osteochondral fragments, such as steel or bio-absorbable screws, bio-absorbable darts or pins, K-wire, and bony pegs. Recent reports in both acute and chronic cases showed that either absorbable devices or bone pegs were mostly used.^3,4,7,8,26-30^

**Figure 1.**
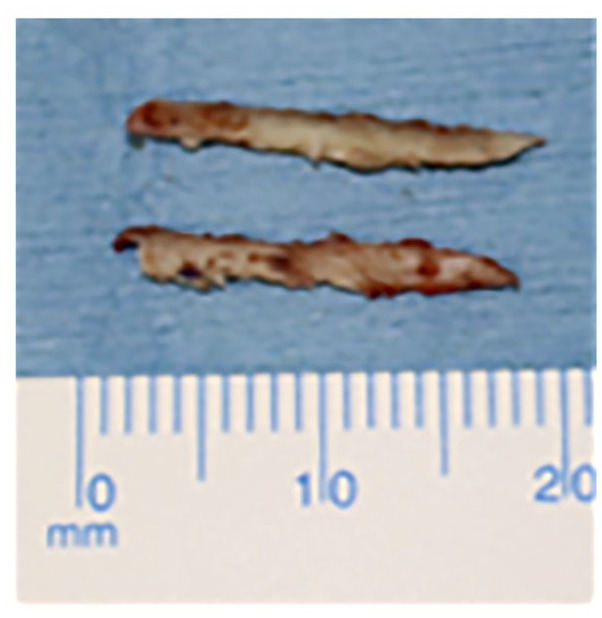
Bone pegs.

Bony pegs are widely used for the fixation of osteochondral lesions or acute osteochondral fractures of various joints.^28,31-35^ Cortical bone pegs have several advantages, such as enhancement of bone union between the osteochondral fragment and its bed and no risk of a foreign body reaction. Cortical bone pegs are harvested as 2.0 to 3.0 mm wide and 18 to 20 mm long from the distal tibia and their tips sharpened (**Fig. 1**). Finally, bone pegs do not have the potential downside of iatrogenic damage from protruding screws.

Bio-absorbable pins have also been used to fix osteochondral fragments. Bio-absorbable pins have the advantage of their relatively simple use and the availability of various diameters and lengths as off-the-shelf products. In addition, the recent trend of all-arthroscopic procedures has encouraged their use.^36^ Potential disadvantages of using bio-absorbable pins compared to cortical bone pegs are inflammatory reactions due to a foreign body reaction.^37,38^ There are several reports regarding progressive osteolysis and cyst formation after PLLA (Poly-L-Lactic Acid) implants in the spine, upper, and lower extremities.^39-42^ A previous report showed that a shallow pin insertion angle induced osteolytic change around pins in OLT because a shallow pin insertion angle might induce micromotion of pins by a high joint compression force.^43^ In addition, pre-operative subchondral bone trabecular deterioration is associated with the incidence of post-operative osteolysis around the PLLA pins,^44^ which may result in the failure of stable fixation. The PLLA pins should therefore be inserted as vertically as possible into the lesion to avoid osteolytic changes around the pins. Bio-absorbable compression screws can provide a larger compression force than bio-absorbable pins and bone pegs and are useful for the fixation of large osteochondral fragments.^45^

## Approach

The surgical approach for fixation can be open through an arthrotomy, osteotomy, or arthroscopic. For lateral lesions, the anterior talofibular ligament and/or the accessory anteroinferior tibiofibular ligament are excised to expose the lesion. Most lateral lesions are exposed with the ankle in full plantar flexion.^8^ However, some cases of lateral OLT may require osteotomy of the anterolateral corner of the tibia and at the distal end of the fibula. For medial OLT, a medial malleolus osteotomy is often performed to obtain good visualization and sufficient working space. The major disadvantage of osteotomy is the potential to negatively impact clinical outcomes due to pain at the osteotomy site and non-union or malunion of the osteotomy site which can lead to osteoarthritis due to abnormal contact loading stress on the joint surface.^46,47^ To overcome this disadvantage, all-inside arthroscopic procedures have been reported. Nakagawa *et al.*^48^ demonstrated an arthroscopic fixation technique using the medial transmalleolar approach. In their technique, the medial transmalleolar portal was made with a 3.0-mm K-wire, and the external cylinder of a 2.7-mm arthroscope was inserted into the bone tunnel. Subsequently, a 2.0-mm K-wire was inserted through the cylinder and drilled into the OLT. A 2.0-mm PLLA pin was inserted through the cylinder into the OLT, and multiple pin fixation was performed by changing the angle of plantar flexion and dorsiflexion of the ankle. It is also reported that 1.5-mm PLLA pins were arthroscopically inserted to fix the osteochondral fragment through a bone tunnel of 2.0-mm diameter at the medial malleolus.^49^ The downside of the transmalleolar fixation of an osteochondral fragment is the damaging to the articular tibial cartilage by creating bone tunnels, which may in turn lead to osteoarthritis and reduced patient outcomes. Recently, Kerkhoffs *et al.*^4^ reported an arthroscopic fixation technique, the LDFF (lift, drill, fill, and fix), which overcomes this limitation. During LDFF, an osteochondral flap is made, keeping the posterior side of the flap intact. The osteochondral lesion is lifted with the use of a chisel (lift). The bone of the osteochondral flap and osteosclerotic area of the lesion bed are debrided and drilled to promote revascularization (drill). The defect is thereafter filled with cancellous bone harvested from the distal tibia (fill). Finally, the osteochondral flap is fixed with a bio-compression screw or multiple chondral darts (fix). The LDFF technique can be used for acute lesions or as an intra-articular non-union repair with bone grafting in chronic lesions and showed good clinical outcomes up to long-term follow-up.^4,36,50^ Kim *et al.*^51^ demonstrated fixation of the OLT using a 3-portal posterior arthroscopic technique. The patient was placed in the prone position, and 2 posterior portals were created. The ankle joint was fully dorsiflexed until posteromedial OLT came into view. The modified posteromedial portal was made just posterior to the posterior colliculus of the medial malleolus, and absorbable pins were inserted through this portal. Although arthroscopic fixation is less invasive, incomplete access to the lesions may result in treatment failure due to inadequate reduction and fixation, especially the posteromedial lesion. For these lesions, an open procedure with good exposure to the talar dome may be considered to prevent union complications.^45^

## Clinical Outcomes

### Clinical Scores

Clinical scores for fixation of OLT are available in 10 studies (**Table 1**). There were 4 open procedures and 4 arthroscopic procedures. In the open procedures, 1 study by Kumai *et al.*^7^ used the criteria of Berndt and Harty^52^ to evaluate clinical outcomes. In the aforementioned study, a good outcome was obtained in 24 ankles, a fair outcome in 3 ankles, and none with a poor outcome. Haraguchi *et al.*^8^ reported a Japanese Society for Surgery of the Foot (JSSF) score of 93.0 ± 6.6 points (range 74-100). Among their study of 45 ankles, 1 failure case was reported, with a JSSF <80 in a 70-year-old patient with a non-traumatic zone 4. Nakasa *et al.* and Park *et al.* evaluated clinical outcomes using the American Orthopaedic Foot & Ankle Society (AOFAS) hind-foot scores and reported 98.6 (range 90-100) points in 18 ankles and 91.1 (range 77-100) points in 25 ankles, respectively. Rikken *et al.* showed the clinical outcomes of open LDFF in 15 ankles of 15 patients at 2-year follow-up. The AOFAS significantly improved from 61 to 95 points, and the numeric rating scale (NRS) for pain at rest and during walking significantly improved at final follow-up.^45^ On post-operative CT imaging, 14 ankles (93.3%) exhibited union of the osteochondral fragment, and there were no complications apart from 1 patient with a fragment non-union.

**Table 1.** Summary of the Clinical Outcomes of the Fixation Procedure.

Author, y	Number of Patients, Age	Lesion Size, Type	Follow-up Periods	Fixation Materials	Clinical Outcomes	
					Scores	Images
Kumai *et al.*^7^ 2002	27 ankles in 27 patients (Men 14, women 13) 27.8 y (range 12-62 y)	10.3 × 2.7 mm Berndt and Harty classification stage 2; 18 ankles, stage 3: 8 ankles, stage 4: 1 ankle	7 y (range, 2-19 y)	Bone pegs	The criteria of Berndt and Harty Good 24 ankles, fair 3 ankles One ankle with a poor radiological result required arthroscopic synovectomy 3 y after surgery	Radiographs or CT Good (complete bone union) 22 ankles, fair (incomplete bone union) 2 ankles, poor (no change or collapse or depression) 3 ankles
Kerkhoffs *et al.*^4^ 2016	7 ankles in 7 patients (Men 6, women 1) 17 y (range 14-58 y)	9.6 ± 3.2 mm (coronal), 15.7 ± 3.0 mm (sagittal), 6.7 ± 1.4 mm (deep) Scranton classification stage 3: 5 ankles, stage 4: 2 ankles	12 ± 0.6 mo	Bio-compression screws Chondral darts	AOFAS 63 ± 9.7 to 99 ± 1.6 points NRS pain at rest 2.9 ± 1.9 to 0.1 ± 0.4, pain with walking 7.6 ± 0.5 to 0.1 ± 0.4	Radiographs Five patients showed remodeling and bone ingrowth
Reilingh *et al.*^5^ 2018	14 ankles in 14 patients (Men 5, women 9) 17 y (range 16-18 y)	13 ± 2 mm (sagittal), 9 ± 2 mm (coronal), 6 ± 3 mm (deep) Berndt and Harty classification stage 2: 2 ankles, stage 3: 12 ankles	12 mo	Bio-compression screws Chondral darts	AOFAS 66 ± 10.1 to 89 ± 17.0 points NRS pain at rest 2.1 ± 1.8 to 0.9 ± 1.3, pain with running 7.4 ± 1.9 to 2.5 ± 3.1	CT a depressed subchondral bone: 4 ankles, a flush subchondral bone plate: 10 ankles
Nakasa *et al.*^22^ 2019	18 ankles in 17 patients (Men 10, women 7) 20.1 y (range 14-56 y)	107.7 mm^2^ (range 69.4-182.5 mm^2^) Anderson’s classification stage 3: 16 ankles, stage 4: 2 ankles	18.9 mo (range 12-48 mo)	PLLA pins	AOFAS 72.1 (68-82) to 98.6 (90-100) points	MRI BML on coronal image: 195.5 mm^2^ to 99.5 mm^2^, BML on sagittal image: 247.6 mm^2^ to 131.65 mm^2^, De Smet criteria; incorporation: fair 1 ankle, good 17 ankles
Lambers *et al.*^50^ 2020	27 ankles in 25 patients (Men 14, women 11) 17 y (range 11-63 y)	8 ± 2.2 mm (coronal), 14 ± 2.8 mm (sagittal), 7 ± 3.1 mm (depth) Berndt and Harty classification stage 2: 6 ankles, stage 3: 21 ankles	27 mo (range 18-43 mo)	Bio-compression screws Chondral darts	FAOS; 67 ± 20 to 86 ± 22 for pain, 66 ± 18 to 63 ± 19 for other symptoms, 87 ± 22 to 95 ± 18 for activities of daily living, 40 ± 20 to 70 ± 22 for sport, 22 ± 17 to 53 ± 27 for quality of life NRS during running 7.8 to 2.9, during walking 5.7 to 20, in rest 2.3 to 1.2 SF-36 physical component scale 42.9 ± 9.2 to 50.1 ± 7.7, mental component scale 55.7 ± 6.0 to 49.8 ± 12.0 One patient received BMS 2 y after the initial LDFF due to unsatisfactory results	CT 81% showed a flush subchondral bone plate and 92% of OCD showed union
Haraguchi *et al.*^8^ 2020	45 ankles in 44 patients (Men 18, women 26) 30.0 ± 15.7 y (range 12-79 y)	51.2 mm^2^ (5-147 mm^2^), 7.0 ± 2.6 mm (range 2-15 mm) (coronal), 9.0 ± 3.4 mm (range 2-17 mm) (sagittal) Berndt and Harty classification stage 2 or 3	2.1 y (1-9 y)	Bone pegs	JSSF score 63.5 ± 17.9 (range 28-90) to 93.0 ± 6.6 (range 74-100) points, 1 ankle failure (0.02%) Two patients underwent arthroscopic synovectomy during follow-up period	Radiographs Good (complete bone union) 28 ankles, fair (incomplete bone union) 10 ankles, poor (no change or collapse or depression) 7 ankles
Park *et al.*^53^ 2020	25 ankles in 25 patients (Men 15, women 10) 19.6 y (range 11-34 y)	11.2 mm (5-20 mm) (coronal), 10.4 mm (7-18 mm) (sagittal), 5.2 mm (3-10 mm) (deep) Hepple’s classification stage 2a: 9 (36%), stage 3: 14 (56%), stage 4: 2 (8%)	22 mo (range 12-35 mo)	bone peg	AOFAS 70.6 (range 44-78) to 91.1 (range 77 -100) points VAS 6.3 (range 4-8) to 1.6 (range 0-5)	CT or MRI Good (complete bone union) 19 ankles, fair (incomplete bone union) 6 ankles
Nakasa T *et al.*^16^ 2022	36 ankles in 34 patients (Men 16, women 18) 21.5 y (range 13-59 y)	104.4 ± 35.0 mm^2^ (49.1-182.5 mm^2^)	22.8 ± 11.5 mo (range 12-60 mo)	PLLA pins	AOFAS 71.1 (68-82) to 97.3 (90-100) points	MRI One ankle (3%) showed depressed articular surface
Rikken QGH *et al.*^36^ 2023	20 ankles in 18 patients (Men 9, women 9) 24.2 y	9.4 ± 2.5 mm (coronal), 13.8 ± 2.9 mm (sagittal), 7.0 ± 2.2 mm (depth)	82.9 mo	Bio-compression screws Chondral darts	FAOS; 66.5 (54.0-79.5) to 94.4 (83.3-100) for pain, 69.5 (52.0-75.0) to 71.4 (57.1-84.0) for other symptoms, 90.5 (74.5-97.0) to 98.5 (97.1-100) for activities of daily living, 40.0 to 80.0 (60.0-100) for sport, 22.0 (13.0-34.5) to 56.3 (50.0-68.8) for quality of life NRS during running 8.0 (6.0-10.0) to 2.0 (0.0-4.5), during walking 7.0 (5.0-8.0) to 0.0 (0.0-1.5), in rest 2.5 (1.0-3.0) to 0.0 (0.0-0.0) SF-36 physical component scale 43.3 (35.8-51.1) to 45.1 (41.8-47.8), mental component scale 57.1 (53.5-60.8) to 37.4 (35.4-39.5)	
Rikken QGH *et al.*^45^ 2023	15 ankles in 14 patients (Men 7, women 7) 24 y (range 14-46)		24 mo	Bio-compression screws Chondral darts	AOFAS 61 to 95 points NRS pain at rest 4 to 0, during walking 7 to 0	14 of 15 ankles (93.3%) showed union at 1-y CT scans

AOFAS = American Orthopaedic and Foot Ankle Society; NRS = Numeric Rating Scale; FAOS = Foot and Ankle Outcome Score (FAOS); SF-36 = Short Form-36; JSSF = Japanese Society for Surgery of the Foot; VAS = visual analog scale; BMS = bone marrow stimulation; LDFF = lift; drill, fill, and Fix; BML = bone marrow lesion.

Other studies demonstrated the clinical outcome of all-inside arthroscopic procedures from 1 institution. Kerkhoffs *et al.* reported clinical outcomes of the LDFF technique with a mean AOFAS score of 99.0 ± 1.6 points in 7 ankles. Lambers *et al.*^50^ reported the mid-term outcomes of the LDFF technique in 27 ankles using the Foot and Ankle Outcome Score (FAOS) and the Short Form-36 (SF-36) with a mean follow-up period of 27 months. They showed that FAOS at the final follow-up was 86 ± 22 for pain, 63 ± 19 for other symptoms, 95 ± 18 for activities of daily living, 70 ± 22 for sport, and 53 ± 27 for quality of life. The SF-36 physical component scale improved from 42.9 ± 9.2 to 50.1 ± 7.7, and the mental component scale decreased from 55.7 ± 6.0 to 49.8 ± 12.0. In a long-term follow-up series, 20 ankles that underwent LDFF were evaluated at a mean follow-up of 7 years (minimum 5 years).^36^ This study found that the median NRS during walking, running, and in rest remained stable over time. The FAOS, similarly, remained stable post-operatively on all subscales. In addition, the long-term procedure survival rate of arthroscopic LDFF was observed to be 87%.

Two studies compared the clinical outcomes between fixation and microfracture. Reilingh *et al.*^14^ reported that there was no difference in clinical outcomes between LDFF and BMS at 1-year follow-up, but the subchondral restoration after LDFF was significantly superior to BMS. Nakasa *et al.*^16^ demonstrated the clinical outcomes of fixation were superior to those of BMS even for lesions sized <100 mm^2^, while clinical scores in the fixation group remained stable in the longer follow-up compared to a decrease in the BMS group.

With respect to the reported complications following fixation among the 10 evaluated studies, Park *et al.*^53^ reported that 1 patient had medial pain due to synovitis at the osteotomy site, and an arthroscopic synovectomy was performed when the screws were removed. In the study by Haraguchi *et al.*,^8^ a 70-year-old patient with a non-traumatic zone-4 lesion resulted in failure, and 2 patients required additional surgeries of arthroscopic shaving of the rough cartilage at the peripheral rim of the lesion due to residual symptom. The study by Kumai *et al.*^7^ reported that 1 ankle with a medial lesion showed a poor radiological result, and arthroscopic synovectomy was performed after 3 years due to pain during sporting activities. In the report of open LDFF by Rikken *et al.*, symptomatic hardware removal was performed in 8 patients, among whom 2 patients underwent additional osteophyte and soft-tissue impingement removal. A revision procedure, by means of a Talar OsteoPeriostic grafting from the Iliac Crest (TOPIC) autologous bone grafting, for a recurrent OLT and non-union of the fragment was performed in 1 patient.^45^

### Image Analysis

**Figure 2.**
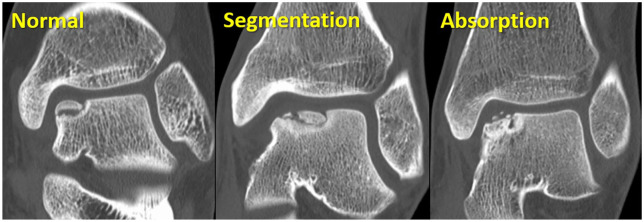
Three types of osteochondral fragment, normal, segmentation, and absorption on the CT images according to the previous report.^24^

One year after surgery or final follow-up, image analyses using plain radiography, CT, or MRI were performed in the evaluated studies. Kumai *et al.*^7^ used the following criteria: complete bony union on plain radiographs or CT classified as good, bony union that was improved but incomplete as fair, and no change compared with the pre-operative state, collapse, or depression of the lesion as poor. The aforementioned study showed good results in 22 ankles (82%), fair in 2 ankles (7%), and poor in 3 ankles (11%). Three ankles radiologically rated as poor exhibited progressive collapse or depression of the osteochondral fragment. The mean interval between the onset of symptoms and surgery in these cases was 5 years. One ankle with poor radiological results underwent arthroscopic synovectomy 3 years after surgery. Kerkhoffs *et al.*^4^ showed that 5 of 7 defects showed remodeling and bone ingrowth after fixation on the final radiograph. In the report by Haraguchi *et al.*,^8^ good radiographic healing outcomes were observed in 28 ankles, fair in 10 ankles, and poor in 7 ankles. The aforementioned authors showed that there was no significant relationship between the post-operative JSSF scores and radiographic outcomes. Nakasa *et al.*^23^ demonstrated that the osteochondral fragment was classified into 3 types on CT images: normal, segmentation, and absorption. Normal indicates a normal contour of the subchondral bone of the osteochondral fragment. Segmentation indicated an almost normal subchondral bone plate, but the fragment was segmented. Absorption showed that bone absorption had progressed, and the subchondral bone plate disappeared in the osteochondral fragment (**Fig. 2**). On MRI at 1-year follow-up, the bone marrow lesion area in the absorption lesion type was significantly larger than in the other 2 lesion type groups. As for incorporation on MRI, all ankles in the normal and segmentation groups showed good results, but 1 ankle in the absorption group displayed fair results. The normal and segmentation types showed good congruity in all ankles, whereas the absorption type exhibited slight irregularities in the 2 ankles. Lambers *et al.*^50^ found that 81% of ankles (21/26) showed a flush subchondral bone plate, and 92% (24/26) of OLT showed a union of the osteochondral fragment at 1-year CT scan. In a report by Park *et al.*,^53^ used either CT or MRI and the radiological criteria by Kumai *et al.*,^7^ good outcomes were observed in 19 ankles (76%), fair in 6 ankles (24%), and poor outcomes in none.^7^ Reilingh *et al.*^5^ compared the post-operative CT images of arthroscopic BMS and LDFF, which showed a significant difference in the healing of the subchondral bone plate favoring fixation. In their report, a depressed subchondral bone plate was observed in 11 patients, and 3 patients had a flush subchondral bone plate after arthroscopic BMS, while a depressed subchondral bone plate was found in 4 patients and a flush subchondral bone plate in 10 patients after arthroscopic LDFF. Based on previous studies, the complete bony union can be expected in a minimum of 76% of cases, regardless of the fixation method.

### Second-Look Arthroscopy

One study reported results from second-look arthroscopy following fixation. Nakasa *et al.*^23^ examined the relationship between biopsy specimens at initial surgery and second-look arthroscopic findings. They showed that there was no significant difference in the ICRS scale (range 7-12 points) for the type of osteochondral fragment, normal, segmentation, and absorption.^24^ The mean Mankin score of the biopsy specimens was 5.7 of 13 points (range 2-9 points), and there was no significant correlation between the Mankin score and ICRS score of second-look arthroscopy. Good clinical outcomes in the short-term follow-up can be obtained in OLT within ICRS grade 2 and moderate degeneration of cartilage, but it is unclear whether good clinical outcomes in the long-term follow-up can be sustained in these ankles.

## Clinical Cases

In this study, we provide 4 cases, 2 from the Japanese and 2 from the Amsterdam perspective, highlighting the different approaches and fixation options for OLT, including a failure case. These cases are provided as examples for clinicians as to what sort of lesions and situations they might encounter.

### Case 1

**Figure 3.**
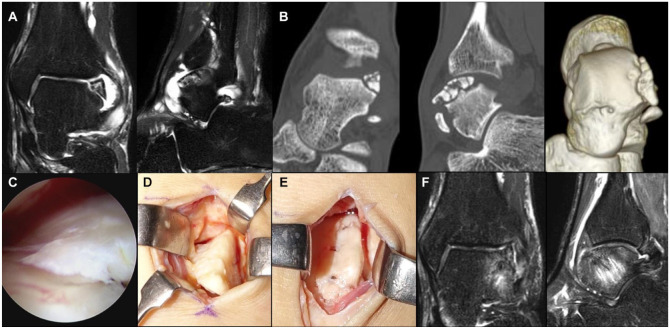
Case 1. (**A**) Pre-operative magnetic resonance imaging (MRI). (**B**) Pre-operative computed tomography (CT) images. (**C**) Arthroscopic findings. The smooth surface of the osteochondral fragment is observed. (**D**) Macroscopic findings. (**E**) After fixation using bio-absorbable pins. (**F**) Post-operative MRI at 1 year after surgery.

A 32-year-old male presented due to left ankle pain. He had sprained his left ankle 20 years ago and was treated conservatively. His complaints of pain and joint swelling gradually increased. Imaging revealed an OLT on the lateral side of the talus (**Fig. 3A**). The osteochondral fragment was segmented but the subchondral bone plate remained, which suggested that the osteochondral unit was maintained. During surgery, a smooth articular cartilage surface on the osteochondral fragment was observed arthroscopically (**Fig. 3B**). Fixation of the osteochondral fragment was therefore performed. The OLT was exposed through a mini-open approach by extending the anterolateral portal, and the lesion bed was debrided (**Fig. 3C**). The osteochondral fragment was formed to match the bed and subsequently fixed using bio-absorbable pins (**Fig. 3D**). One year after surgery, the patient had no symptoms and the JSSF scale improved to 100 points compared to 68 points pre-operatively. A post-operative MRI revealed a union of the osteochondral fragment and its bed (**Fig. 3E**).

### Case 2

**Figure 4.**
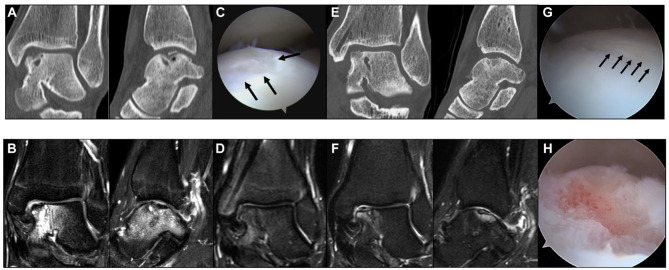
Case 2. (**A**) Pre-operative CT images. (**B**) Pre-operative MRI. (**C**) Arthroscopic findings. Arrows indicate fibrous tissues. (**D**) Post-operative MRI at 1 year after surgery. (**E**) Post-operative CT at 6 years after surgery. (**F**) Post-operative MRI at 6 years after surgery. (**G**) Arthroscopic findings. Arrows indicate a fissure between the osteochondral fragment and surrounding normal cartilage. (**H**) After resection of the osteochondral fragment.

A 17-year-old male presented due to bilateral ankle pain. Bilateral medially located OLT were observed on advanced imaging. OLT in the left ankle showed a cystic lesion in the bed and bone component in the osteochondral fragment was scarce (**Fig. 4A** and **B**). For both lesions, fixation of the osteochondral fragment was performed. During arthroscopy of the left ankle, the surface of the osteochondral fragment was found to be smooth and comprised of cartilaginous and fibrous tissues (**Fig. 4C**). The biopsy specimen of the cartilage in the osteochondral fragment showed 9 points of Mankin score. Bone grafting to the cystic lesions and fixation using bio-absorbable pins were performed through a medial malleolus osteotomy approach. The post-operative course was good and an MRI at 1 year post-operatively showed good contour and bone union between the osteochondral fragment and its bed (**Fig. 4D**). Six years later, the patient presented due to pain and locking of the left ankle joint. An MRI showed detachment of the cartilage fragment, but CT images revealed that the subchondral bone was repaired well (**Fig. 4E** and **F**). During surgery, an unstable osteochondral fragment was observed during arthroscopy (**Fig. 4G**) and arthroscopic excision of the fragment was performed (**Fig. 4H**). Hereafter, the complaints were completely resolved. This case suggests that the osteochondral fragment with scarce bony components may not fuse biologically and would benefit from additional bone grafting.

### Case 3

**Figure 5.**
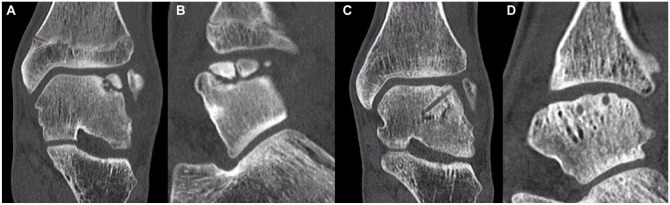
Case 3. Pre-operative CT with coronal (**A**) and sagittal (**B**) views, showing an anterolateral osteochondral fragment. The fragment was comprised of 2 smaller fragments and was likely traumatized previously. The lesion measured anteriorposteriorly: 25 mm, medial-laterally: 11 mm, and depth: 8 mm. One-year post-operative CT with coronal (**C**) and sagittal (**D**) views, showing the union of the fragment and a good bony integration. It should be mentioned that the radiolucency in the talus is the bio-absorbable screw.

A 14-year-old male, playing football at a national level, who presented 5 months after an inversion injury during a sliding tackle. The patient presented with pain during weightbearing and a feeling of instability, rendering him unable to play sports. Upon physical examination, a noticeable pain in the lateral ankle and no positive drawer test were observed. Coronal (**Fig. 5A**) and sagittal (**Fig. 5B**) view of a CT scan found an anterolateral osteochondral fragment. The fragment was comprised of 2 smaller fragments and likely traumatized previously. The lesion measured anterior-posteriorly (AP) 25 mm, medial-laterally (ML) 11 mm, and depth 8 mm. Access to the lesion was obtained through a lateral arthrotomy, without the need for an additional osteotomy. Both fragments were successfully and individually fixed with bio-absorbable screws according to the LDFF technique,^45^ without compromising the articular cartilage structure. At the 1-year visit, the patient was playing football at his pre-injury level and did not report any pain. CT examination showed a union of the fragment and a good bony integration (**Fig. 5C** and **D**) at 1-year post-operative CT. It should be mentioned that the radiolucency in the talus is the bio-absorbable screw. These can remain visible post-operatively and can, in some cases, persist. Clinically, these have no implications as the fragment has achieved union and the screw is stable without protrusion.

### Case 4

**Figure 6.**
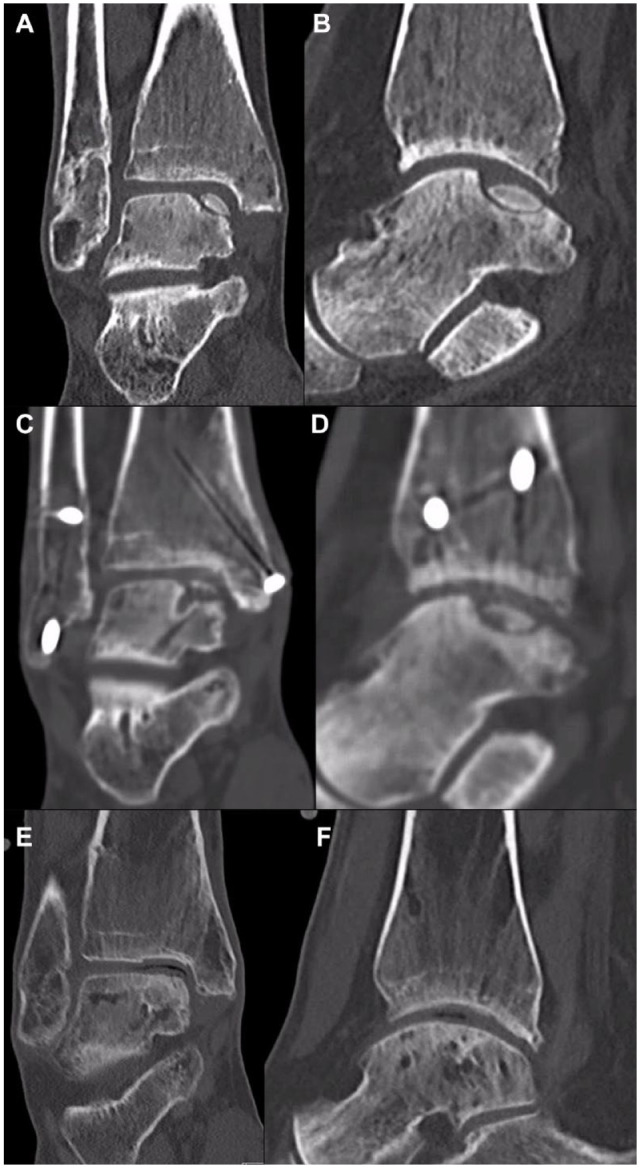
Case 4. Pre-operative CT with coronal (**A**) and sagittal (**B**) views, showing a pseudoarthrosis of the distal fibula and an osteochondral fragment on the posterior-medial talar dome. The fragment measured anteriorposteriorly: 15 mm, medial-laterally 10 mm, and depth: 6 mm. The 8.5 months post-operatively after sustaining an inversion injury the CT with coronal (**C**) and sagittal (**D**) views showed a consolidated medial malleolus osteotomy, consolidated pseudoarthrosis repair of the distal fibula but a non-union of the osteochondral fragment without fragment displacement or signs of hardware complications. Hereafter, the patient underwent a medial TOPIC procedure. The results were satisfactory with a good incorporation of the graft and osteotomy union at the 3-year follow-up as outlined on the 3-year post-operative CT with coronal (**E**) and sagittal (**F**) views.

A 43-year-old woman, who was referred 7 months after a conservatively treated distal fibular fracture with persistent ankle pain, both on the lateral ankle as well as deep in the medial side of the ankle. The pain was exacerbated during walking, rendering her unable to work. Upon physical examination, poor strength and active stability, as well as pain over the distal fibula and medial talar dome, were observed. A CT scan (**Fig. 6A** and **B**) found a pseudoarthrosis of the distal fibula and an osteochondral fragment on the posterior-medial talar dome. The fragment measured 15 mm AP, 10 mm ML, and 6 mm in depth. The patient underwent a pseudoarthrosis repair of the distal fibula and LDFF of the medial osteochondral fragment with 1 bio-absorbable screw placed centrally and 1 chondral dart placed anteriorly to provide rotational stability. Access to the lesion was obtained through a medial malleolus osteotomy. The post-operative trajectory was normal and uncomplicated, up until 8 months post-operatively when the patient sustained an inversion injury. The patient presented 8.5 months post-operatively at our clinic for CT examination, which showed a consolidated medial malleolus osteotomy, consolidated pseudoarthrosis repair of the distal fibula but a non-union of the osteochondral fragment without fragment displacement or signs of hardware complications (**Fig. 6C** and **D**). Clinically, there were no signs of a (low-grade) infection. As a salvage operation, the patient underwent a TOPIC procedure (**Fig. 6E** and **F**).^54^

## Future Perspective

Promising results for fixation of OLT have been reported, but more evidence is needed to improve the evidence-based and patient-centered treatment regimen. Although it has been reported that fixation has better post-operative clinical outcomes when compared to BMS,^14,16^ there are no comparative studies with fixation and other procedures for OLT, such as autologous matrix-induced chondrogenesis (AMIC) or autologous chondrocyte implantation (ACI). Since good clinical outcomes of these procedures have been reported, comparisons of mid- or long-term clinical outcomes of these procedures with fixation are required, which may help to improve surgical indications for OLT.^55,56^ In addition, the limitations of fixation are still unclear due to lack of evidence. From previous reports, fixation is performed on OLT with a relatively good bony stock and articular cartilage (up to ICRS grade 2) of the osteochondral fragment, resulting in good clinical outcomes. It is necessary to analyze the clinical outcomes in a large number of fixations, including failure cases to determine the limitation of fixation in the future. In image analyses, there are several cases with incomplete union or non-union, and it should be elucidated which factors may have predisposed these patients to non-union and whether these ankles will exhibit progressive collapse or depression of the osteochondral fragment. According to a report by Kumai *et al.*,^7^ the mean interval between the onset of symptoms and surgery in the ankles rated as poor was 5 years, which suggested that deterioration of the subchondral bone would progress. Another factor to consider in future work is the subchondral bone status and any additional interventions to improve healing. As the subchondral bone plays a crucial role in maintaining articular cartilage homeostasis, interventions are required to improve the subchondral bone condition and promote bone healing. Recently, augmentation by means of biologic adjuvants such as platelet-rich plasma, bone marrow aspirate concentrate, and stem cells has been reported.^57^ Finally, it is important to study the effect of additional bone grafting in fixation procedures of OLT on the union rate. Biologic adjuvants could enhance bone and cartilage healing due to several growth factors, stem cells, and anti-inflammatory effects.

## Conclusion

Recent evidence revealed that fixation for OLT can obtain good clinical results in short- to mid-term follow-up. Although further investigations are needed to evaluate the clinical outcomes in long-term follow-up cases and limitations of the procedure, the fixation procedure should be considered as the first-line treatment in fixable OLT because it can restore the native articular surface with hyaline cartilage.
